# Supplementary material for: SMC modulates ParB engagement in segregation complexes in streptomyces
Source: Nat Commun. 2025 Oct 9;16:8999. doi: 10.1038/s41467-025-64044-3 (PMC12511625; doi:10.1038/s41467-025-64044-3)
Supplement: Supplementary file 1 — Supplementary Information [file 41467_2025_64044_MOESM1_ESM.pdf]

**SUPPLEMENTARY INFORMATION**

# SMC modulates ParB engagement in segregation complexes in *Streptomyces*

**Katarzyna Pawlikiewicz[1], Agnieszka Strzałka[1], Michał Majkowski[2], Julia Duława-Kobeluszczyk[1], Marcin J. Szafran[1], Dagmara Jakimowicz[1*]**

[1] Department of Molecular Microbiology, Faculty of Biotechnology, University of Wroclaw, Wroclaw, Poland

[2] Advanced Imaging and Cytometry laboratory, Faculty of Biotechnology, University of Wroclaw, Wroclaw, Poland

**SUPPLEMENTARY FIGURES** ........ 2

**SUPPLEMENTARY TABLES** ........ 12

**BIBLIOGRAPHY** ........ 15

## SUPPLEMENTARY FIGURES

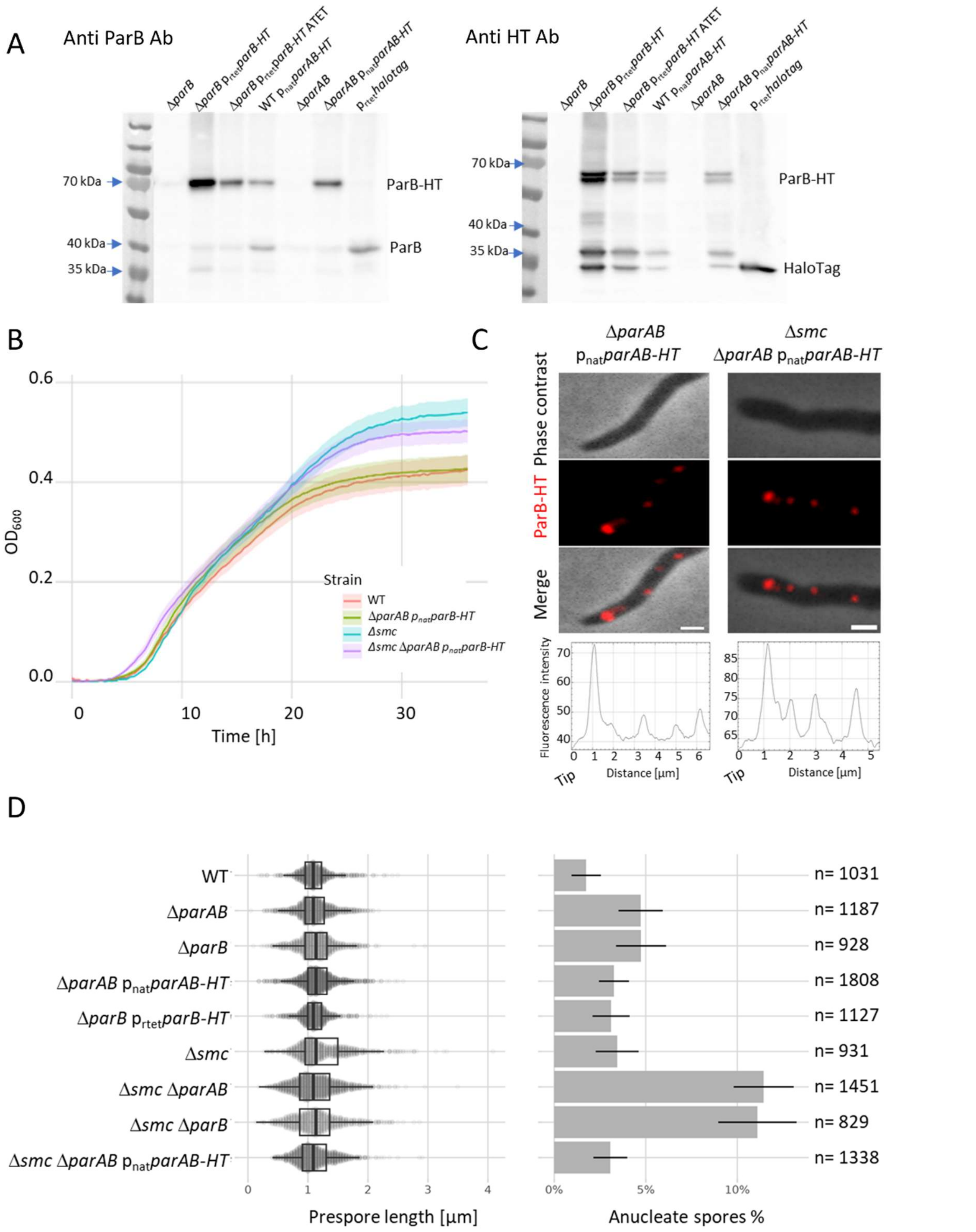


**Supplementary Figure 1. Verification of ParB-HT producing *S. venezuelae* strains. A.** Western blotting analyses of ParB-HT levels in modified *S. venezuelae* strains (as indicated): Δ*parB* (MD020), Δ*parB* p$_{rtet}$*parB-HT* (KP009) cultured without and with addition of anhydrotetracycline(ATET, 100 ng/ml), WT p$_{nat}$*parAB-HT* (KP005), Δ*parAB* p$_{nat}$*parAB-HT* (KP006) and WT p$_{rtet}$*halotag* (KP003), Left panel – anti ParB antibody, right panel – anti HaloTag antibody. The positions of ParB, ParB-HT and Halotag are indicated.

Note that *parB-HT* gene under the control of $p_{rtet}$ promoter is downregulated in the presence of ATET - reverse TetR repressor ($TetR_{rv}$) binds to *tetO* sites in presence of ATET [1]. The images show representative of two independent experiments. **B.** Growth curves of wild type *S. venezuelae* strain (WT) and Δ*smc* strain (TM010) compared to growth curve of Δ*parAB* $p_{nat}$*parAB-HT* (KP006) and Δ*smc* Δ*parAB* $p_{nat}$*parAB-HT* (KP007). **C.** Representative images showing ParB-HT complexes stained with TMR in young vegetative cells (5 hours of growth) of Δ*parAB* $p_{nat}$*parAB-HT* (KP006) and Δ*smc* Δ*parAB* $p_{nat}$*parAB-HT* (KP007) strain. TMR - red, phase contrast – gray. Scale bar – 1 μm. The lower panel shows the analyses of fluorescence intensity. **D.** Analysis of septation defects (prespore length) - left panel and chromosome segregation defects (% of anucleate spores) – right panel in modified *S. venezuelae* strains. Boxplots show the median value with 1st and 3rd quartile as box boundaries and whiskers extending to mean +/- 1.5 * IQR. Number of analysed spores (n) is indicated.

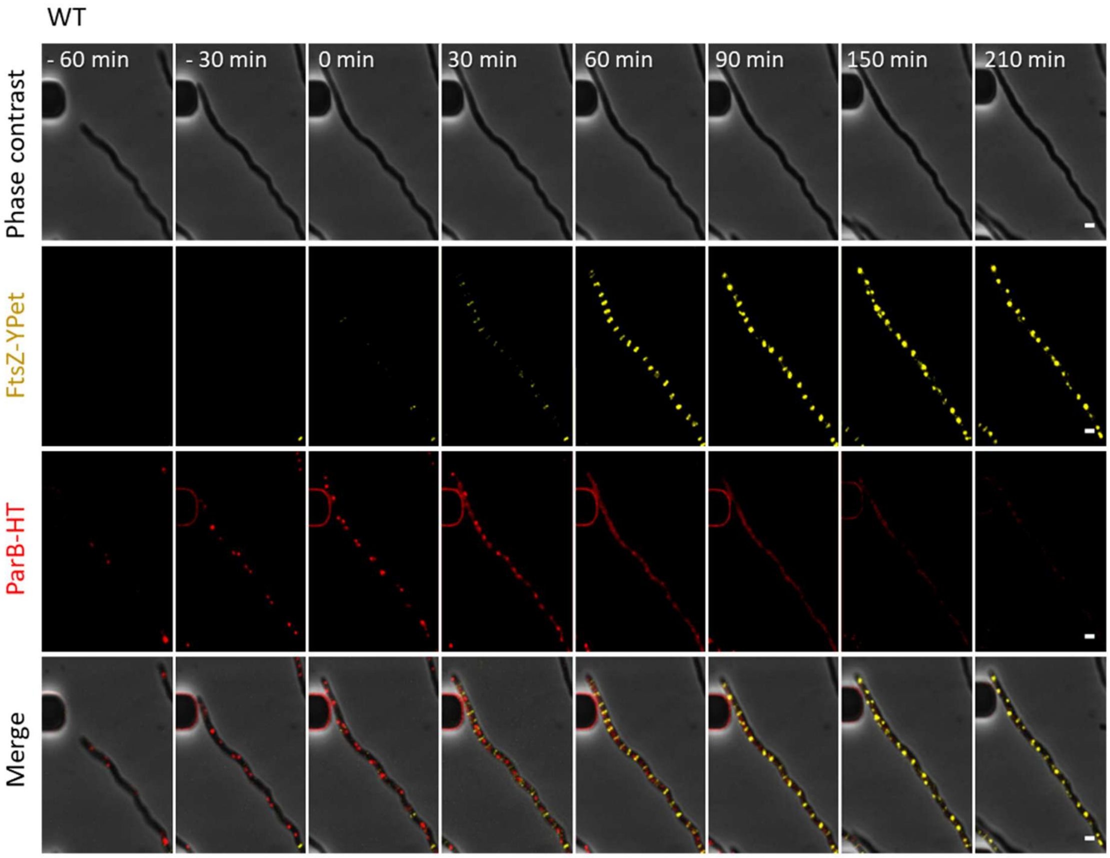


**Supplementary Figure 2. Time lapse analyses of ParB-HT complexes and septation during sporogenic development of *S. veneuelae*.** Representative images from time lapse analysis **(Supplementary Movie 1)** of sporogenic development of Δ*parB* $p_{rtet}$*parB-HT ftsZ-ypet* (KP011) strain with fluorescence of ParB-HT stained with Janelia Fluor-549 (red), FtsZ-YPet fluorescence (yellow), phase contrast (grey) and all three channels overlaid. Time 0 is the time of hyphal cell growth arrest, scale bar – 1 μm.

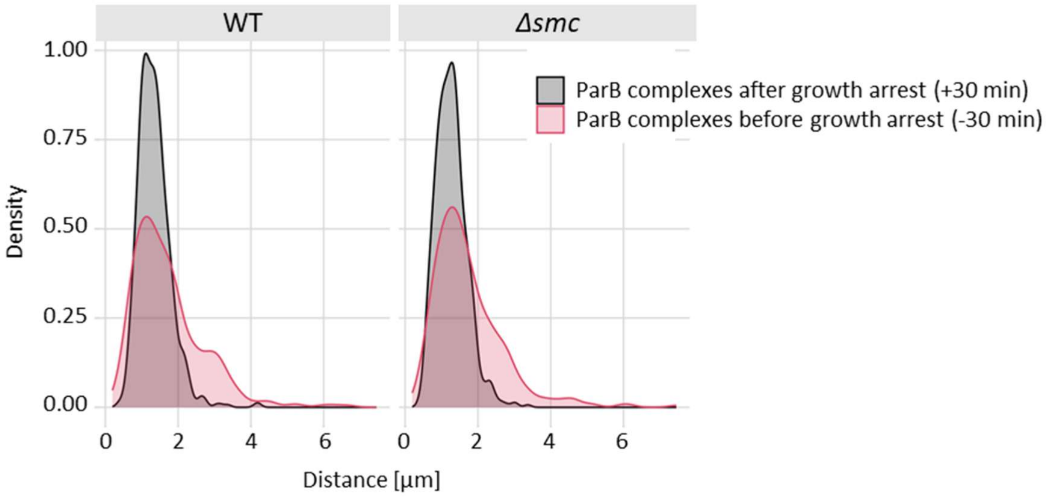


**Supplementary Figure 3.** Analysis of the distances between the ParB-HT complexes in extending hyphal cells (30 min before growth arrest) and after growth arrest in the Δ*parAB* $p_{nat}$*parAB-HT*, KP006 and in Δ*smc*Δ*parAB* $p_{nat}$*parAB-HT*, KP007 strain. Data were collected in 6 independent experiments for 35 hyphae (before growth arrest) and 73 hyphae (after growth arrest) of KP006 strain and 46 hyphae (before growth arrest) and 78 hyphae (after growth arrest) of KP007 strain.

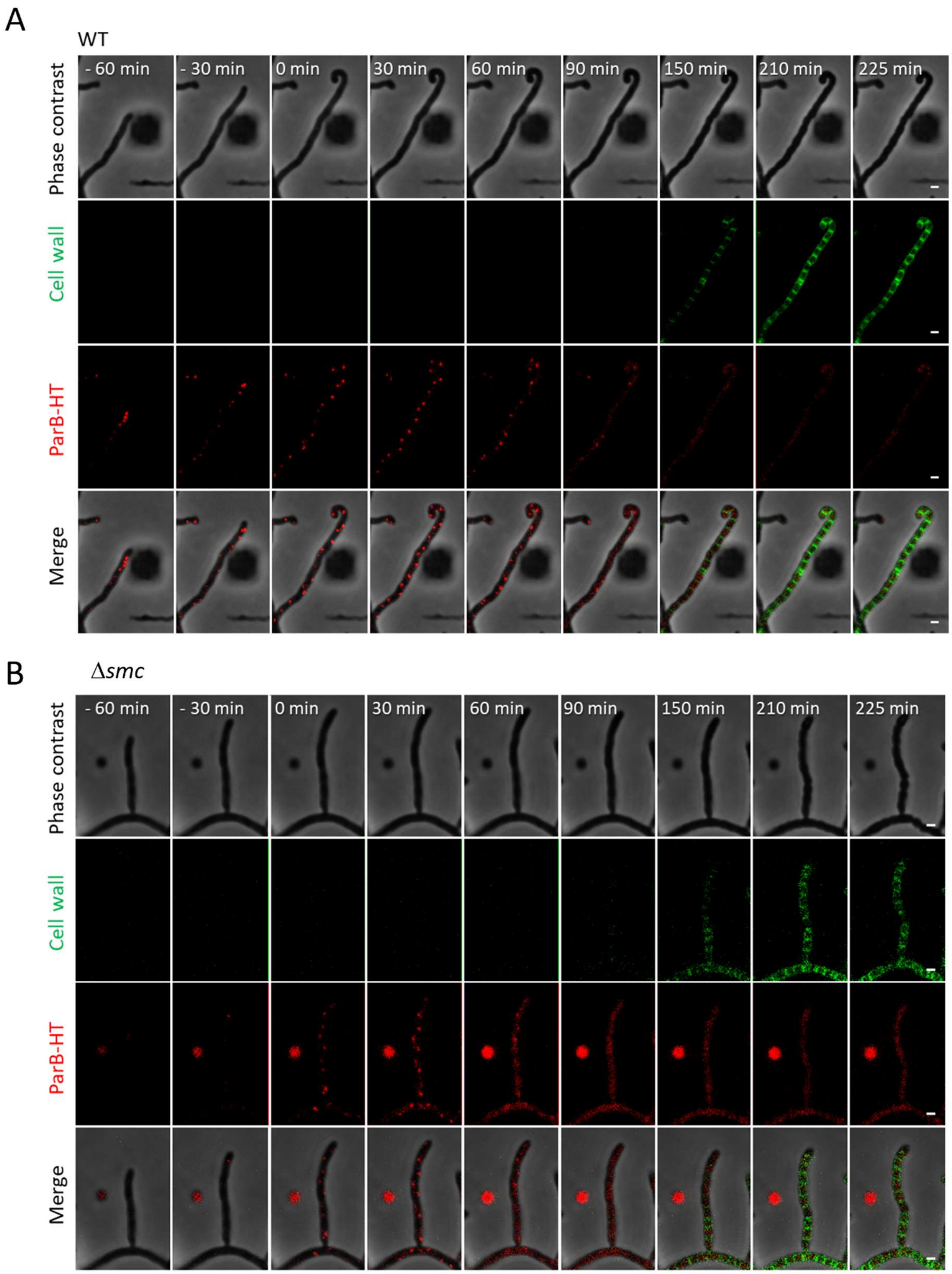


**Supplementary Figure 4. Time lapse analyses of ParB-HT complexes and septation in *S. venezuelae* wild type and *smc* deletion background A. and B** Representative images from time lapse analysis of sporogenic development of wild type control Δ*parAB* p$_{nat}$*parAB-HT*, KP006 **(A) (Supplementary Movie 2)** and Δ*smc* Δ*parAB* p$_{nat}$*parAB-HT*, KP007 strain **(B) (Supplementary Movie 3)** with fluorescence of ParB-HT stained with Janelia Fluor-549 (red), fluorescence of NADA green-stained septa (green), phase contrast (grey) and all three channels overlaid. Scale bar – 1 μm.

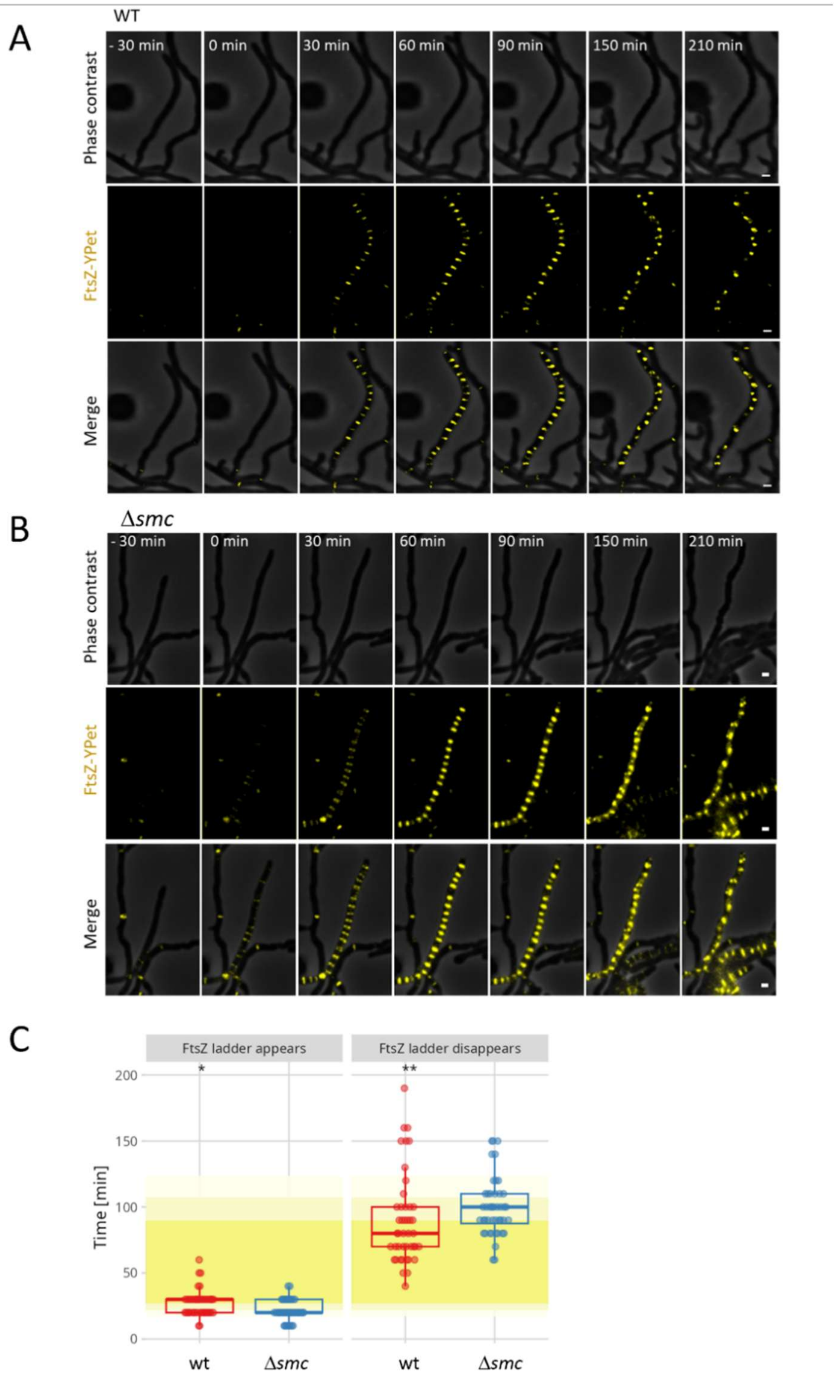


**Supplementary Figure 5. Time lapse analyses of Z-ring timing in *S. venezuelae* wild type and *smc* deletion background. A and B** Representative images from time lapse analysis of sporogenic development of wt *ftsZ-ypet* (MD100) **(A) (Supplementary Movie 4)** and Δ*smc ftsZ-ypet* strain (TM004) **(B) (Supplementary Movie 5)** showing FtsZ-YPet fluorescence (yellow), phase contrast (grey), and the overlay of two channels. Scale bar – 1 μm. **C.** Analysis of the timing of Z-rings assembly and disassembly in relation to sporogenic cell growth arrest. Yellow shading shows the mean lifetime of Z-rings. Boxplots show the median value with 1st and 3rd quartile as box boundaries and whiskers extending to mean +/- 1.5 * IQR. Data shown in panel C were collected in 4 independent experiments for 44 hyphae of MD100 and TM004 strains, statistical analyses were performed using two-sided Wilcoxon test. *p-values:* Z rings appear: 0.01, Z rings disappear: 0.009.

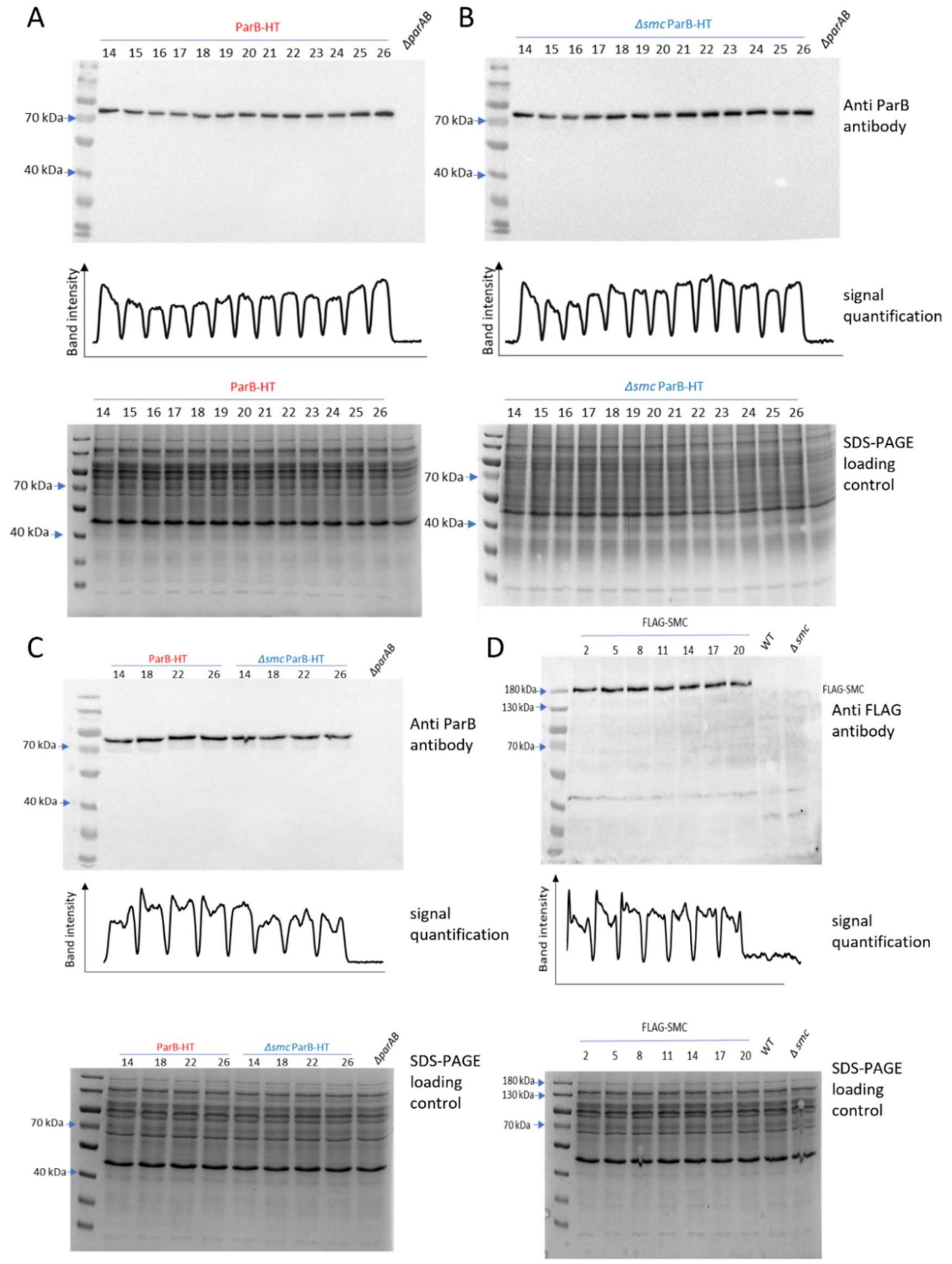


**Supplementary Figure 6. Levels of ParB-HT in wild type and *smc* deletion background and levels of FLAG-SMC during the life cycle of *S. venezuelae*.** Western blotting detection of ParB-HT in cell lysates of the Δ*parAB* $p_{nat}$*parAB-HT*, KP006 **(A)** and in Δ*smc*Δ*parAB* $p_{nat}$*parAB-HT,* KP007 strain **(B)** from different time points during sporogenic development (14 h to 26 h of culture) **C.** Western blotting detection of ParB-HT - comparison of ParB-HT levels in Δ*parAB* $p_{nat}$*parAB-HT*, KP006 and Δ*smc*Δ*parAB* $p_{nat}$*parAB-HT,* KP007, (A-C anti ParB antibody) **D.** Western blotting detection of FLAG-SMC in cell lysates of the TM017 strain from different time points of the life cycle (2 h to 20 h of culture, as indicated) using anti FLAGtag antibody. **A-D A**nalyses of band signal intensity and loading control – cell lysates analysed in SDS PAGE gel stained with Coomasie brilliant blue are shown as below Western blotting panels. All the panels show representative of at least two independent experiments.

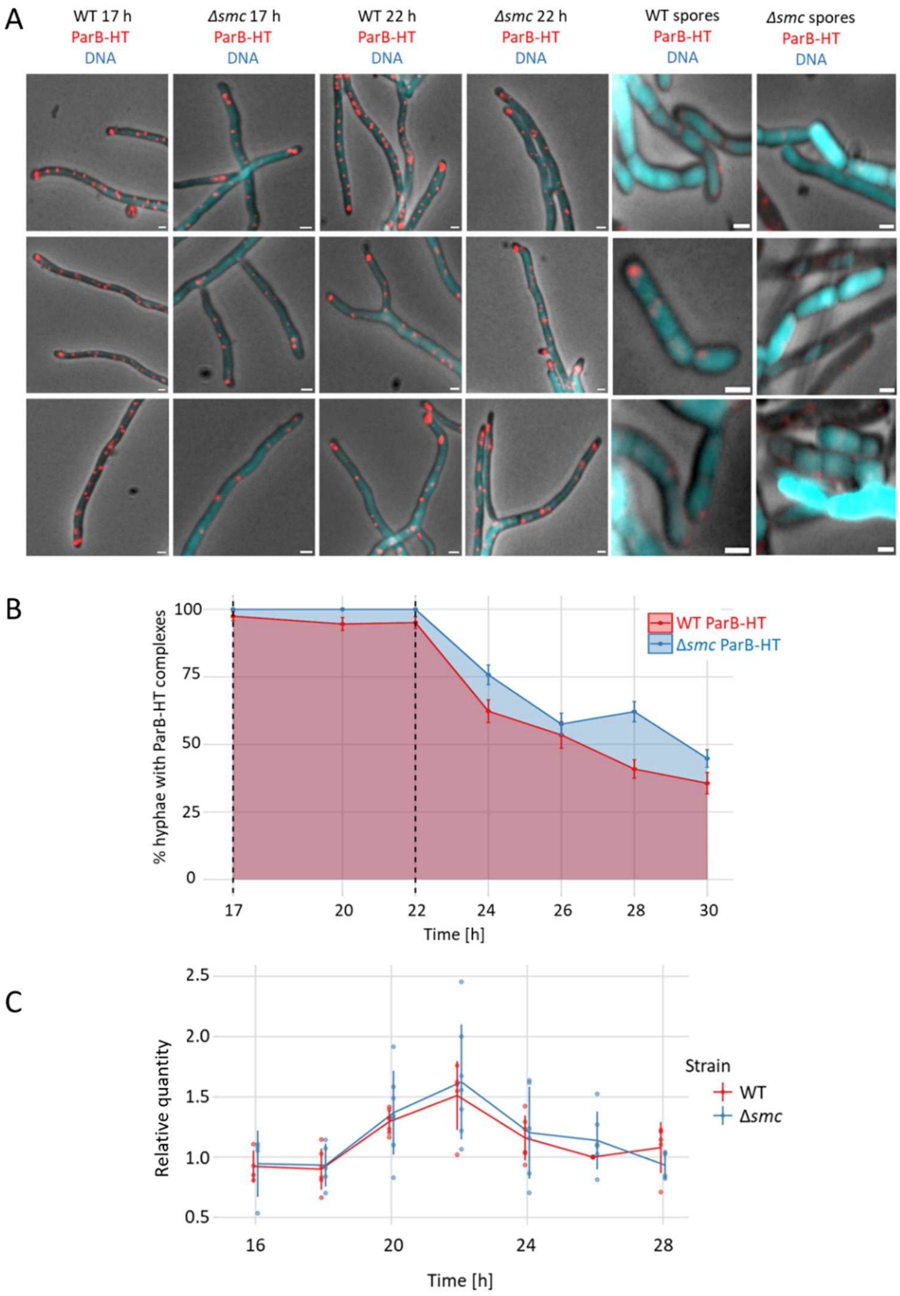


**Supplementary Figure 7. The progress of sporogenic development of strains producing ParB-HT in wild type and Δ*smc* background determined by the percentage of hyphae showing ParB complexes.** **A.** Representative images of hyphal cells of the wild type control and Δ*smc* strains (Δ*parAB* $\text{p}_{\text{nat}}$*parAB-HT*, KP006 and Δ*smc*Δ*parAB* $\text{p}_{\text{nat}}$*parAB-HT* KP007, respectively) from three time points of sporogenic development. ParB-HT was stained with TMR Direct Ligand (red) and DNA was stained with Hoechst 33342 (blue) overlaid on phase contrast (grey). Scale bar – 1 μm. **B.** The percentage of hyphae showing ParB complexes (either regularly or irregularly spaced) in wild type control and Δ*smc* strains (Δ*parAB* $\text{p}_{\text{nat}}$*parAB-HT*, KP006 (350-802 hyphae) and Δ*smc*Δ*parAB* $\text{p}_{\text{nat}}$*parAB-HT* KP007 (187-915 hyphae), respectively) at subsequent time points of sporogenic development. Error bars show 95% confidence intervals. **C**. Efficiency of chromosome replication determined by marker frequency analysis – *ori:ter* ratio during sporogenic development of wild type control and Δ*smc* strains (Δ*parAB* $\text{p}_{\text{nat}}$*parAB-HT*, KP006 and Δ*smc*Δ*parAB* $\text{p}_{\text{nat}}$*parAB-HT* KP007, respectively). Error bars show standard deviation.

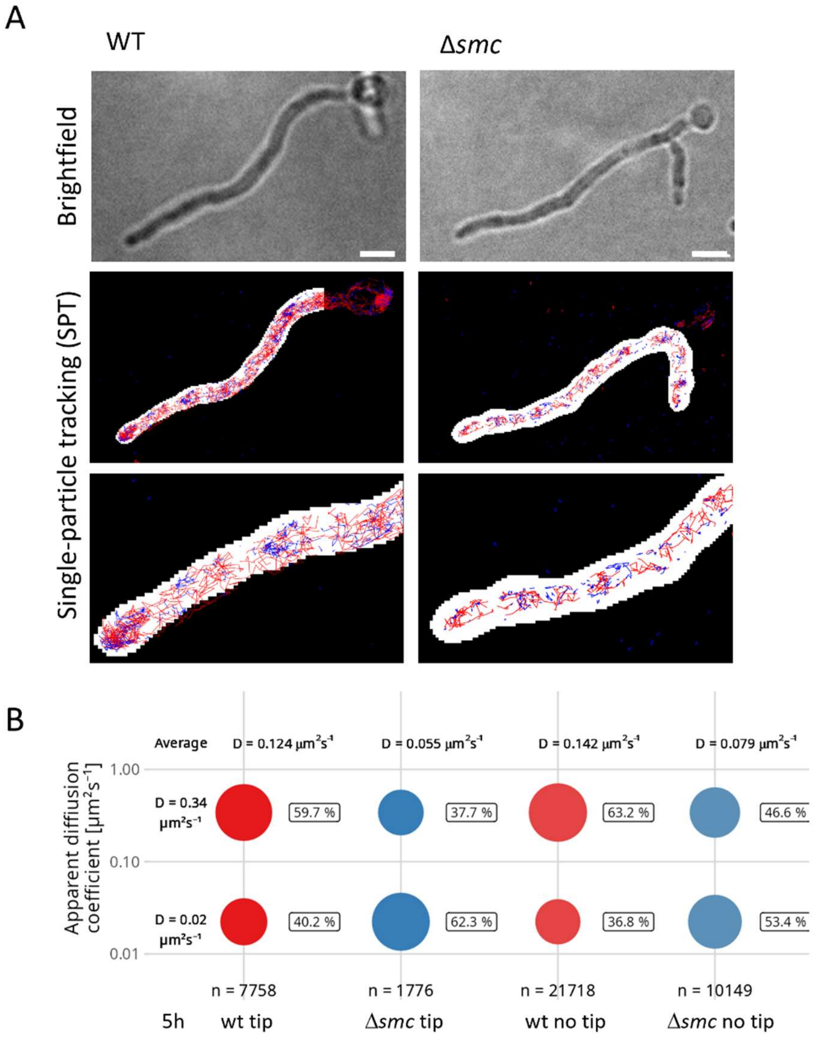


**Supplementary Figure 8. Single molecule tracking of ParB-HT. A.** Representative hyphae of the wild type control and Δ*smc* strains (Δ*parAB* $p_{nat}$*parAB-HT*, KP006 and Δ*smc*Δ*parAB* $p_{nat}$*parAB-HT* KP007, respectively). Top images - brightfield images of analysed hyphae, middle and bottom images - ParB-HT tracking labelled accordingly to mean speed – red: fast moving, blue: slow moving molecules. Bottom images are scaled up. Scale bar 2 μm **B**. Comparison of ParB-HT mobility in tip-proximal and tip-distal region of vegetative cell. Percentage of ParB-HT tracks with low (D=0.02 $\mu m^2/s$) and high (D = 0.34 $\mu m^2/s$) diffusion coefficient in proximity to the tip (<3 μm, tip) and in the distance greater than 3 μm from the tip (no tip) of the wild type control (KP006) and Δ*smc* (KP007) strains

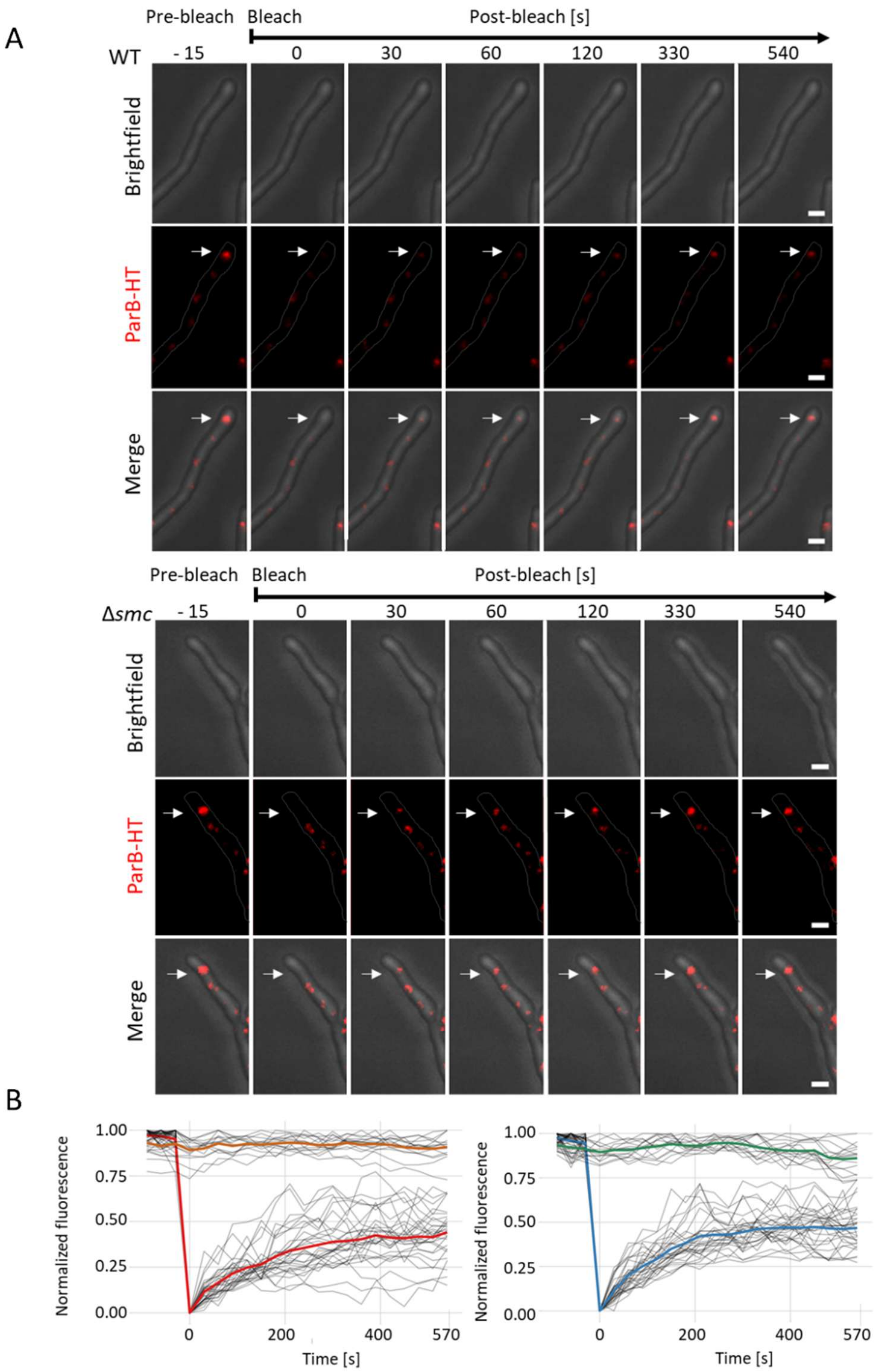


**Supplementary Figure 9. FRAP analysis of ParB-HT complexes in early vegetative cell of *S. venezuelae* the wild type and Δ*smc* strain. A** Representative images showing the photobleaching of the ParB-HT complex stained with Janelia Fluor-549 in young vegetative cells of the wild type control **(Supplementary Movie 6)** and Δ*smc* **(Supplementary Movie 7)** strains (Δ*parAB* $p_{nat}$*parAB-HT*, KP006 and Δ*smc*Δ*parAB* $p_{nat}$*parAB-HT* KP007, respectively), ParB-HT fluorescence (red) brightfield channel (grey) and overlay of both channels **B.** Fluorescence recovery analysis. Intensity of normalized bleached fluorescence plotted against the time of analyses compared to control fluorescence signal (not bleached) in the wild type control (left panel) and Δ*smc* strain (right panel). 22 complexes were analysed in the wild type and Δ*smc* background (Δ*parAB* $p_{nat}$*parAB-HT*, KP006 and Δ*smc*Δ*parAB* $p_{nat}$*parAB-HT,* KP007).

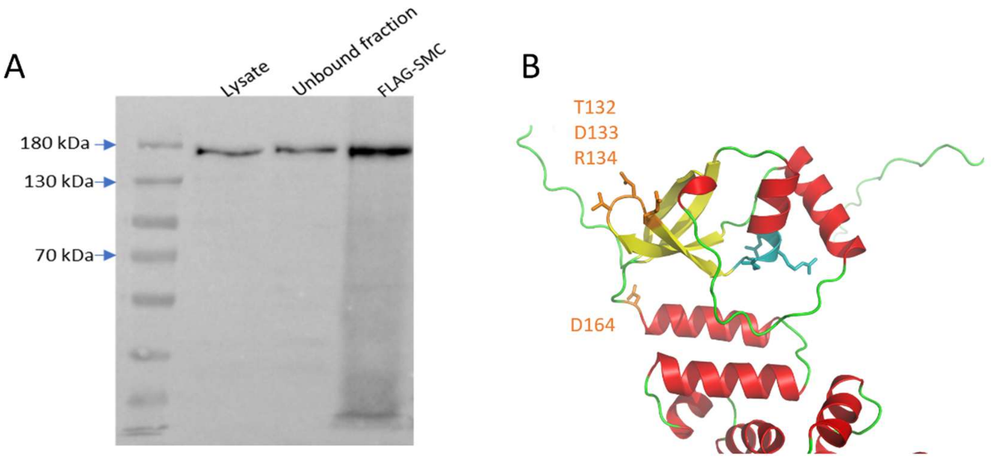


C

```
B.sub  25   EIKIADLRPNPYQPRKHFDDEALAELKESVLQHGILQPLIVRKS-LKGYDIVAGERRFRA   83
            E+ +   + PNP QPR+ FD++ALAEL  S+ + G+LQP++VR++    Y+++ GERR+RA
S.ven  87   ELPLDSITPNPRQPREVFDEDALAELVTSIKEVGLLQPVVVRRAGTDRYELIMGERRWRA  146
ParBSMC-                                                 AAT

B.sub  84   AKLAGLDTVPAIVRELSEALMREIALLENLQREDLSPLEEAQAYDSLLKHLDLTQEQLAK  143
             + AGL+ +PAIVR+   +   +   ALLENL R  L+PLEEA AYD LLK     T +QLA
S.ven  147  CREAGLERIPAIVRDTEDEKLLLDALLENLHRAQLNPLEEAAAYDQLLKDFHCTHDQLAD  206
ParBSMC-                      R
```

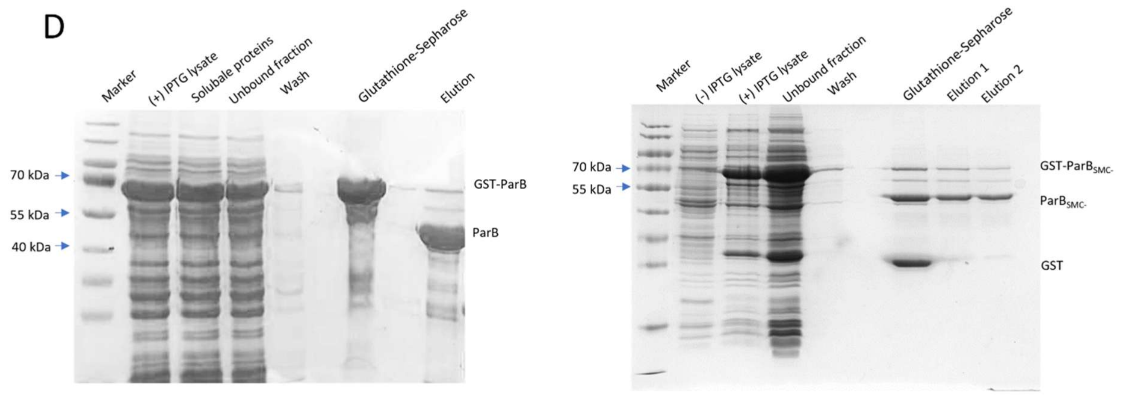


**Supplementary Figure 10. Protein preparation for CTPase activity assay. A. Western blotting analysis of FLAG-SMC purification from *S. venezuelae*. A.** FLAG-SMC was immunoprecipitated from 20-hour culture of TM017 strain using magnetic beads coated with anti-FLAG® BioM2 antibody and released with 3XFLAG peptide. FLAG-SMC was detected using anti-FLAG antibody in the TM017 cell lysate, fraction not bound to magnetic beads and fraction released from magnetic beads with 3XFLAG peptide (FLAG-SMC). **B.** fragment of *S. venezuelae* ParB structure featuring the loop involved in ParB-SMC interactions (Alpha fold AF-A0A5P2A7Y7-F1) visualised with PyMOL[2–4]**C.** Sequence comparison of *B. subtilis* and *S. venezueale* ParB. The amino acids involved in the interaction with SMC in *B. subtilis* and the corresponding region in *S. venezuelae* ParB are shown in orange. **D.** Purification of ParB (lef panel) and $ParB_{SMC-}$ (right panel) cleaved from a GST fusion.

## SUPPLEMENTARY TABLES

### Supplementary Table 1. Bacterial strains used in this study

| Name | Description | Source |
|---|---|---|
| | ***E. coli*** | |
| **DH5α** | F-, Φ80dlacZM15, recA1, *endA1, gyrA96, thi-1,hsdR17, (rk-, mk+), supE44, relA1, deoR, (lacZY*AargF) *U169* | Lab stock |
| **ET12567/pUZ8002** | *dam13::TN9, dcm6, hsdM, hsdR, recF134, zjj201::TN10, galK2, galT22, ara14, lacY1, xyl5, leuB6, thi1, tonA31, rpsL136, hisG4, tsx78, mtli, glnV44*, F-, ($Cml^R$ ) pUZ8002: tra, $Kan^R$,RP4 23 | [5] |
| | ***S. venezuelae*** | |
| **WT** | Wild type strain *Streptomyces venezuelae,* NRRL culture collection deposited under NRRL number B-65442, by NZ_CP018074.1 | Kind gift from Prof. Mark Butt-ner John Innes Centre, Nor-wich, UK[6] |
| **TM010 (Δ*smc*)** | NRRL B-65442 *Δsmc* :: scar | [7] |
| **TM017 (*FLAG-smc*)** | *smc* :: *FLAG-smc* | [7] |
| **TM004 (Δ*smc*; *ftsZ-ypet*)** | TM010 attBΦC31 :: pKF351 *ftsZ-ypet* , $Apr^R$ | [7] |
| **MD002 (*ΔparB::apra*)** | WT *ΔparB::apra*, $Apr^R$ | [8] |
| **MD020 (Δ*parB*)** | WT *ΔparB*::scar | [8] |
| **MD030 (Δ*parAB*)** | WT *ΔparAB*::scar | [8] |
| **MD100 (*ftsZ-ypet*)** | WT attBΦC31::pKF351, $Apr^R$ | [8] |
| **KP003 (WT $p_{rtet}$*halotag*)** | WT attBϕBT1::pKP03 (pSS170 $p_{rtet}$*halotag*), $Hyg^R$ | This work |
| **KP004 (Δ*smc*Δ*parAB*)** | TM010 Δ*parAB*::*apra*, $Apr^R$ | This work |
| **KP020 (Δ*smc*Δ*parB*)** | TM010 *ΔparB::apra,* $Apr^R$ | This work |
| **KP005 (WT$p_{nat}$*parAB*-HT)** | WT attBϕBT1::pKP05 (pSS170 $p_{nat}$*parAB*-HT), $Hyg^R$ | This work |
| **KP006 (Δ*parAB*$p_{nat}$*parAB*-HT)** | MD030 attBϕBT1::pKP05 (pSS170 $p_{nat}$*pa-rAB*-HT), $Hyg^R$ | This work |
| **KP007 (Δ*smc*Δ*parAB*$p_{nat}$*parAB*-HT)** | KP004 attBϕBT1::pKP05 (pSS170 $p_{nat}$*pa-rAB*-HT), $Apr^R$, $Hyg^R$ | This work |
| **KP008 (WT $p_{rtet}$*parB*-HT)** | WT attBϕBT1::pKP08 (pSS170 $p_{rtet}$*parB*-HT), $Hyg^R$ | This work |
| **KP009 (Δ*parB* $p_{rtet}$*parB*-HT)** | MD020 attBϕBT1::pKP08 (pSS170 $p_{rtet-}$*parB*-HT), $Hyg^R$ | This work |
| **KP010 (Δ*parB::apra* $p_{rtet}$*parB*-HT)** | MD002 attBϕBT1::pKP08 (pSS170 $p_{rtet}$*parB*-HT), $Apr^R$, $Hyg^R$ | This work |
| **KP011 (Δ*parB* $p_{rtet}$*parB*-HT *ftsZ-ypet*)** | KP009 attBϕC31::pKF351, attBϕBT1::pKP08 (pSS170 $p_{rtet}$*parB*-HT), $Apr^R$, $Hyg^R$ | This work |

**Supplementary Table 2. Constructs used in this study**

| Name | Description | Source |
|---|---|---|
| **pSS170 (pIJ10770)** | pMS825 derivative, integrative vector attBΦBT1, *ori* pBR322, *oriT* (RP4) $Hyg^R$ | Kind gift from dr Susan Schlimpert, John Innes Centre, Norwich, UK [9] |
| **pSS170 *halotag*** | pSS170 derivative, carrying *halotag* gene, att-BΦBT1, *ori* pBR322, *oriT* (RP4) $Hyg^R$, | [10] |
| **pSS170$p_{erm}$ *halotag*** | pSS170 derivative, carrying *halotag* gene under the control of the $p_{erm}$ promotor, attBΦBT1, *ori* pBR322, *oriT* (RP4), $Hyg^R$, | [10] |
| **pTC-28S15-0X $p_{smyc}$-*tetR*$_{rv}$$p_{tcp}$*topA*$_{Ms}$** | A non-integrative plasmid carrying *M. smegmatis topA* gene under the control of the $p_{tcp830}$ promoter and the *tetR*$_{rv}$ gene under the control of the $p_{myc}$ promoter [11], *ori* pBR322, $Kan^R$, | [12] |
| **pKP03 (pSS170 $p_{rtet}$*halotag*)** | pSS170 derivative, carrying *halotag* under the control of the $p_{tcp830}$ promoter with an $RBS_{topA,}$ and the *tetR*$_{rv}$ gene under the control of the $p_{s14}$ promoter [11], attBΦBT1, *ori* pBR322, *oriT* (RP4), $Hyg^R$ | This work |
| **pKF351** | pIJ6902 derivative, carrying *ftsZ-ypet* gene under the control of a native promoter $p_{ftsZ}$, attBΦC31, *oriT* (RP4), $Apr^R$ | [8] |
| **pKP05 (pSS170 $p_{nat}$*parAB-HT*)** | pSS170 derivative, carrying *S. venezuelae parAB* genes under the control of the native *parAB* promoter, with *parB* in fusion with the *halotag* gene, attBΦBT1, *ori* pBR322, *oriT* (RP4), $Hyg^R$ | This work |
| **pKP08 (pSS170 $p_{rtet}$*parB-HT*)** | KP03 derivative, carrying *parB-halotag* gene under the control of the $p_{tcp830}$ promoter, an $RBS_{topA,}$ and the *tetR*$_{rv}$ gene under the control of the $pS_{14}$ promoter attBΦBT1, *ori* pBR322, *oriT* (RP4), $Hyg^R$ | This work |
| **pCRISPR $p_{tcp}$ $RBS_{topA}$** | Derivative of pCRISPR (Tong et al., 2015), carrying *dcas9* gene controlled by $p_{tcp}$ promoter and $RBS_{topA}$, ApmR, *ori* pBR322, *oriT* (RP4) | Gongerowska-Jac M. , unpublished |
| **Sv-4-A09 Δ*parB::apra*** | SuperCos-1 cosmid Sv-4-A09 derivative, *parB*::*apr-oriT-FRT*, $Amp^R$, $Kan^R$, $Apr^R$ | [8] |
| **Sv-4-A09 Δ*parAB::apra*** | SuperCos-1 cosmid Sv-4-A09 derivative, *parAB*::*apr-oriT-FRT* cassette, , $Kan^R$ $Apr^R$ | [8] |
| **pBSK*parS*** | pBSKc carrying a fragment of *parAB* promoter from *S. coelicolor* (365 bp), containing a single *parS* site, $Amp^R$ | This work |
| **pBSK*parS*$_{mut}$** | pBSKc carrying a fragment of *parAB* promoter from *S. coelicolor* (365 bp), containing a mutated *parS* site, $Amp^R$ | This work |
| **pGEX-Sv*parB*** | pGEX-6P-2 containing *gst* fused to S. *venezuelae parB* gene, $Amp^R$ | This work |
| **pGEX-Sv*parB*$_{SMC-}$** | pGEX-6P-2 containing *gst* fused to S. *venezuelae parB* gene with mutations resulting in the TDR132-134AAT and D164R exchange, $Amp^R$ | This work |

**Supplementary Table 3. Oligonucleotides used in this work**

| Oligonucleotide | Sequence 5' → 3' | Application |
|---|---|---|
| **KP_38Fw** | GTACCGCGGATCGTGCTCATGTTCTCTCCCTT GAATTCTAAT | Construction of pKP03 (pSS170 p$_{rtet}$*halotag*) |
| **KP_38Rv** | TCGATGATCATATGAGAGAATCTAAGCTT TACG TAGACCTACGCCTTGACCTTG | |
| **KP_39Fw** | ATATGATCATCGATTCGCGACTTAAGCCTAGGC CACCTGACCGCACGCCCGCAA | |
| **KP_39Rv** | TCGGATCCATCGTTATTCCTAGGCATATGTCGCT CTTCTCTCCGGGAT | |
| **KP_37Fw** | AAGGGGATGATAAGTTTATCAAGCTTCCATGGTC ATTAGGAGCCGCTCT | |
| **KP_37Rv** | TTAGAATTCAAGGGAGAGAACATGAGCACGATC CGCGGTAC | |
| **KP_38.2Rv** | TCATATGAGAGAATCTAAGCTTTACGTAGACCTA CGCCTTGAC | |
| **KP_37BHTRv** | GCTCACTCAACTGGATCCCCTCCTGTCGCTCTT CTCTCCGGGATA | Construction of pKP05 (pSS170 p$_{nat}$*parAB*-HT) and pKP08 (pSS170 p$_{rtet}$*parB*-HT) |
| **KP_66Fw** | GATTCGCGACTTAAGCATTCTAGAGGGGATCGT CGATCCGCtctcc | |
| **KP_43Rv** | TCGGATCCATCGTTATTCCTAGGCATCTCGAGG CCCTCGGCGTCCTCGGCGTT | |
| **KP_43BHTFw** | TATCCCGGAGAGAAGAGCGACATGGAGGGGAT CCAGTGAGTGAGCGA | |
| **pSSseq_Fw** | AGGATCTTCACCTAGATCCTTTTGGT | Verification of the pSS170 plasmid insert |
| **pSSseq_Rv** | GCCAGTGGTATTTATGTCAACACCGC | |
| **pSv_parAB-kont3_Fw** | CCCGCGAGATCGCCCTG | Verification of *parA* and/or *parB* deletion |
| **pSv_parAB-kont4_Rv** | ATCCAGGCCTCCTTCTCCA | |
| **gyr2_Fw** | GCTCCGCTATCACAAGATCA | Marker frequency analysis: amplification of *oriC* poximal fragment of the S. *venezuelae* chromosome 3,958,240 – 3,958,322 |
| **gyr2_Rv** | ACAGGAAGGTCAGCAGCAG | |
| **arg3_Fw** | CACCTGCGGATCTACAAGC | Marker frequency analysis: amplification of "arm" fragment of the *S. venezuelae* chromosome 1,320,274 – 1,320,351 |
| **arg3_Rv** | CCACTCCGACATCTCCTTG | |
| **parA_fw** | CGCAAGCTTGCGCCGCGGCCGACCCCCGC | Amplification of the *parAB* promoter region from *S. coelicolor* |
| **parAB_rv** | CCGGATCCGACCCGGGTCTGCTCGGGTCGC | |
| **parS_mut fw** | CGGATGTTTCTAGGGAAACACCGC | Mutation of the *parS* site within the *parAB* promoter region from *S. coelicolor* |
| **parS_mut rv** | GCGGTGTTTCCCTAGAAACATCCG | |
| **pGEX_parB_FW** | TCCAGGGGCCCCTGGGATCCGTCGGAGGG-GATCCAGTGAG | Cloning of the *S. venezueale parB* gene to pGEX-6P-2 |
| **pGEX_parB_RV** | TCACGATGCGGCCGCTCGAGTCAGCCCTCG GCGTCCTCGG | |

## BIBLIOGRAPHY


1. Klotzsche, M., Ehrt, S. & Schnappinger, D. Improved tetracycline repressors for gene silencing in mycobacteria. *Nucleic Acids Res* **37**, 1778–1788 (2009).
2. Delano, W. L. The PyMOL Molecular Graphics System. *http://www.pymol.org* https://api.semanticscholar.org/CorpusID:60136037 (2002).
3. Varadi, M. *et al.* AlphaFold Protein Structure Database: massively expanding the structural coverage of protein-sequence space with high-accuracy models. *Nucleic Acids Res* **50**, D439–D444 (2022).
4. Jumper, J. *et al.* Highly accurate protein structure prediction with AlphaFold. *Nature* **596**, 583–589 (2021).
5. Kieser, T., Bibb, M. J., Buttner, M. J., Chater, K. F. & Hopwood, D. A. Practical Streptomyces Genetics. *John Innes Centre Ltd.* 529 (2000) doi:10.4016/28481.01.
6. Gomez-Escribano, J. P. *et al.* Streptomyces venezuelae NRRL B-65442: Genome sequence of a model strain used to study morphological differentiation in filamentous actinobacteria. *J Ind Microbiol Biotechnol* **48**, (2021).
7. Szafran, M. J., Jakimowicz, D. & Elliot, M. A. Compaction and control-the role of chromosome-organizing proteins in Streptomyces. *FEMS Microbiol Rev* **44**, 725–739 (2020).
8. Donczew, M. *et al.* ParA and ParB coordinate chromosome segregation with cell elongation and division during Streptomyces sporulation. *Open Biol* **6**, 150263 (2016).
9. Bush, M. J., Chandra, G., Findlay, K. C. & Buttner, M. J. Multi-layered inhibition of Streptomyces development: BldO is a dedicated repressor of whiB. *Mol Microbiol* **104**, 700–711 (2017).
10. Duława-Kobeluszczyk, J. *et al.* The activity of CobB1 protein deacetylase contributes to nucleoid compaction in Streptomyces venezuelae spores by increasing HupS affinity for DNA. *Nucleic Acids Res* **52**, 7112–7128 (2024).
11. Klotzsche, M., Ehrt, S. & Schnappinger, D. Improved tetracycline repressors for gene silencing in mycobacteria. *Nucleic Acids Res* **37**, 1778–1788 (2009).
12. Szafran, M. J. *et al.* Amsacrine derivatives selectively inhibit Mycobacterial topoisomerase I (TopA), impair M. smegmatis growth and disturb chromosome replication. *Front Microbiol* **9**, (2018).
13. Cobb, R. E., Wang, Y. & Zhao, H. High-Efficiency Multiplex Genome Editing of Streptomyces Species Using an Engineered CRISPR/Cas System. *ACS Synth Biol* **4**, 723–728 (2015).
