# Supplementary material for: SMC modulates ParB engagement in segregation complexes in streptomyces
Source: Nat Commun. 2025 Oct 9;16:8999. doi: 10.1038/s41467-025-64044-3 (PMC12511625; doi:10.1038/s41467-025-64044-3)
Supplement: Supplementary file 3 — Description of Additional Supplementary Files [file 41467_2025_64044_MOESM3_ESM.pdf]

Description of Additional Supplementary Files

File Name: Supplementary Movie 1
Description: **Time lapse analyses of ParB-HT complexes and septation.** Sporogenic development of Δ*parB* p_{rtet}*parB-HT ftsZ-ypet* (KP011) strain with fluorescence of ParB-HT stained with Janelia Fluor549 (red), FtsZ-YPet fluorescence (yellow) overlaid on phase contrast (grey).

File Name: Supplementary Movie 2
Description: **Time lapse analyses of ParB-HT complexes and septation in wild type background.** Sporogenic development of wild type control Δ*parAB* p_{nat}*parABHT*, KP006 strain with fluorescence of ParB-HT stained with Janelia Fluor549 (red), fluorescence of NADA green-stained septa (green) overlaid on phase contrast (grey).

File Name: Supplementary Movie 3
Description: **Time lapse analyses of ParB-HT complexes and septation in *smc* deletion background.** Sporogenic development of Δ*smc* Δ*parAB* p_{nat}*parABHT*, KP007 strain with fluorescence of ParB-HT stained with Janelia Fluor549 (red), fluorescence of NADA green-stained septa (green) overlaid on phase contrast (grey).

File Name: Supplementary Movie 4
Description: **Time lapse analyses of Zring timing in wild type background.** Sporogenic development of wt *ftsZ- ypet* (MD100) showing FtsZ-YPet fluorescence (yellow) overlaid on phase contrast (grey).

File Name: Supplementary Movie 5
Description: **Time lapse analyses of Zring timing in *smc* deletion background.** Sporogenic development of Δ*smc ftsZ-ypet* strain (TM004) showing FtsZ-YPet fluorescence (yellow) overlaid on phase contrast (grey).

File Name: Supplementary Movie 6
Description: **FRAP analysis ParB-HT complexes in early vegetative cell in the wild type background.** Photobleaching of the ParBHT complex stained with Janelia Fluor-549 in young vegetative cells of the wild type control (Δ*parAB* p_{nat}*parABHT*, KP006). ParB-HT fluorescence (red) overlaid on brightfield channel (grey).

File Name: Supplementary Movie 7
Description: **FRAP analysis ParB-HT complexes in early vegetative cell in the Δ*smc* background.** Photobleaching of the ParBHT complex stained with Janelia Fluor-549 in young vegetative cells of the Δ*smc* strain (Δ*smc*Δ*parAB* p_{nat}*parABHT* KP007, respectively). ParB-HT fluorescence (red) overlaid on brightfield channel (grey).
