# Supplementary material for: SMC modulates ParB engagement in segregation complexes in streptomyces
Source: Nat Commun. 2025 Oct 9;16:8999. doi: 10.1038/s41467-025-64044-3 (PMC12511625; doi:10.1038/s41467-025-64044-3)
Supplement: Supplementary file 11 — Reporting Summary [file 41467_2025_64044_MOESM11_ESM.pdf]

Corresponding author(s): Dagmara Jakimowicz
Last updated by author(s): Jul 17, 2025

Reporting Summary

Nature Portfolio wishes to improve the reproducibility of the work that we publish. This form provides structure for consistency and transparency in reporting. For further information on Nature Portfolio policies, see our Editorial Policies and the Editorial Policy Checklist.

Statistics

For all statistical analyses, confirm that the following items are present in the figure legend, table legend, main text, or Methods section.

n/a	Confirmed	
☐	☒	The exact sample size (*n*) for each experimental group/condition, given as a discrete number and unit of measurement
☐	☒	A statement on whether measurements were taken from distinct samples or whether the same sample was measured repeatedly
☐	☒	The statistical test(s) used AND whether they are one- or two-sided<br>*Only common tests should be described solely by name; describe more complex techniques in the Methods section.*
☒	☐	A description of all covariates tested
☐	☒	A description of any assumptions or corrections, such as tests of normality and adjustment for multiple comparisons
☐	☒	A full description of the statistical parameters including central tendency (e.g. means) or other basic estimates (e.g. regression coefficient) AND variation (e.g. standard deviation) or associated estimates of uncertainty (e.g. confidence intervals)
☐	☒	For null hypothesis testing, the test statistic (e.g. *F*, *t*, *r*) with confidence intervals, effect sizes, degrees of freedom and *P* value noted<br>*Give P values as exact values whenever suitable.*
☒	☐	For Bayesian analysis, information on the choice of priors and Markov chain Monte Carlo settings
☒	☐	For hierarchical and complex designs, identification of the appropriate level for tests and full reporting of outcomes
☒	☐	Estimates of effect sizes (e.g. Cohen's *d*, Pearson's *r*), indicating how they were calculated

Our web collection on statistics for biologists contains articles on many of the points above.

Software and code

Policy information about availability of computer code

Data collection

ChIP-seq (provided by Fasteris)
NextSeq Control Software 4.2.0 112
RTA 2.11.3
bcl2fasta2. 17 v2 17.1.14
Microscopy:
AxioVision LE Rel.4.5 ZEN 2012
Zeiss Elyra 7 Superresolution Imaging System (Zeiss)
Leica Stellaris Imaging System (Leica)

Data analysis	Microscopy analysis: R version 4.4.1, R packages: ggplot2 3.5.1, dplyr 1.1.4, Peaks 0.2, findpeaks https://github.com/astrzalka/findpeaks<br><br>SMT analysis: Fiji plugin TrackMate 7.14.0, Oufti, SMTracker v2.0<br><br>Fiji ImageJ 2.14.0/1.54f<br><br>ChIP-seq analysis: bowtie2 2.5.2, samtools 1.19.2, MACS2 2.2.9.1, R package normr 1.30.0<br><br>RT PCR analysis: StepOne Software v 2.0 (Applied Biosystems)

For manuscripts utilizing custom algorithms or software that are central to the research but not yet described in published literature, software must be made available to editors and reviewers. We strongly encourage code deposition in a community repository (e.g. GitHub). See the Nature Portfolio guidelines for submitting code & software for further information.

Data

Policy information about availability of data

All manuscripts must include a data availability statement. This statement should provide the following information, where applicable:

- Accession codes, unique identifiers, or web links for publicly available datasets
- A description of any restrictions on data availability
- For clinical datasets or third party data, please ensure that the statement adheres to our policy

The raw ChIP-seq data generated in this study have been deposited in the ArrayExpress database (EMBL-EBI) under accession code: E-MTAB-14547 [https://www.ebi.ac.uk/arrayexpress/experiments/E-MTAB-14547].
Images and Movies are available in BioImage Archive: Fig. 1 and Fig. S2 under accession code S-BIAD1808, Fig. 2 and S4 under accession code S-BIAD1807, Fig. 3 and FigS8 under accession code S-BIAD1811, Fig. S5 under accession code S-BIAD1813, Fig. S3 under accession code S-BIAD1814 or in Figshare: Fig. 4 data doi: https://doi.org/10.6084/m9.figshare.28730597, Fig. S7 data doi: https://doi.org/10.6084/m9.figshare.28730699, Fig. S1D data: https://doi.org/10.6084/m9.figshare.28730795.

Research involving human participants, their data, or biological material

Policy information about studies with human participants or human data. See also policy information about sex, gender (identity/presentation), and sexual orientation and race, ethnicity and racism.

Reporting on sex and gender	NA
Reporting on race, ethnicity, or other socially relevant groupings	NA
Population characteristics	NA
Recruitment	NA
Ethics oversight	NA

Note that full information on the approval of the study protocol must also be provided in the manuscript.

Field-specific reporting

Please select the one below that is the best fit for your research. If you are not sure, read the appropriate sections before making your selection.

☒ Life sciences ☐ Behavioural & social sciences ☐ Ecological, evolutionary & environmental sciences

For a reference copy of the document with all sections, see nature.com/documents/nr-reporting-summary-flat.pdf

Life sciences study design

All studies must disclose on these points even when the disclosure is negative.

Sample size	The sample size for microscopy experiments was determined according to our protocols for statistical analysis, also used in ealier studies (Szafran et al., 2021, Kois-Ostrowska et al., 2016). ChIP-seq experiment was performed in three biological repeats.
Data exclusions	Microscopy: Only hyphae that remained in focus throughout the experiment were analyzed. SMT - spores, branches and overlapping hyphae were excluded from the analysis.
Replication	The microscopy data including SMT experiments were collected from at least two independent experiments (biological replicates). ChIP-seq data was collected in three biological replicates. Only those experiments that successfully delivered data were regarded as replicates.
Randomization	Not applicable to our study, because our research analyzes bacterial strains not population subgroups.

Blinding	For image analysis automated, non-manual methods were used whenever possible.

Reporting for specific materials, systems and methods

We require information from authors about some types of materials, experimental systems and methods used in many studies. Here, indicate whether each material, system or method listed is relevant to your study. If you are not sure if a list item applies to your research, read the appropriate section before selecting a response.

Materials & experimental systems

n/a	Involved in the study
☐	☒ Antibodies
☒	☐ Eukaryotic cell lines
☒	☐ Palaeontology and archaeology
☒	☐ Animals and other organisms
☒	☐ Clinical data
☒	☐ Dual use research of concern
☒	☐ Plants

Methods

n/a	Involved in the study
☐	☒ ChIP-seq
☒	☐ Flow cytometry
☒	☐ MRI-based neuroimaging

Antibodies

Antibodies used	used for ChiP seq ; rabbit polyclonal anti S. coeliolor ParB<br>other: mouse anti FLAG (Sigma F9291-1MG), mouse anty-FLAG M2- conjugated to magnetic beads (Thermo Scientific A36797) , mouse anti HaloTag (Promega G921A)
Validation	Anti ParB antibodies were affinity purified using recombinant S. venezuelae ParB protein and verified using cell lysates. Rabbit serum containing polyclonal anti-ParB S. coelicolor antibody was obtained at the Institute of Immunology and Experimental Therapy, Polish Academy of Sciences, in Wrocław (Jakimowicz et al., 2002).

Plants

Seed stocks	NA
Novel plant genotypes	NA
Authentication	NA

ChIP-seq

Data deposition

☒ Confirm that both raw and final processed data have been deposited in a public database such as GEO.

☒ Confirm that you have deposited or provided access to graph files (e.g. BED files) for the called peaks.

Data access links<br>*May remain private before publication.*	ChIP-seq data: https://www.ebi.ac.uk/biostudies/arrayexpress/studies/E-MTAB-14547?key=2ca9d6af-d139-43fb-8d3e-f2c0119d64a5
Files in database submission	dparB_PB_1.fastq.gz<br>dparB_PB_2.fastq.gz<br>dsmc_PB_1.fastq.gz<br>dsmc_PB_2.fastq.gz<br>dsmc_PB_3.fastq.gz<br>WT_PB_1.fastq.gz<br>WT_PB_2.fastq.gz<br>WT_PB_3.fastq.gz
Genome browser session<br>(e.g. UCSC)	no longer applicable

Methodology

Replicates	All replicates are biological replicates<br>Number of replicates:<br>WT - 3<br>dsmc - 3<br>dParB - 2
Sequencing depth	All samples: single-end, sequence length 150 bp<br><br>total reads/mapped reads<br>dparB_PB_1 18 515 784/17 315 961<br>dparB_PB_2 17 360 162/16 143 214<br>dsmc_PB_1 22 385 712/21 042 569<br>dsmc_PB_2 25 608 747/24 095 270<br>dsmc_PB_3 22 651 059/21 224 042<br>WT_PB_1 26 064 784/24 842 345<br>WT_PB_2 17 872 810/16 886 230<br>WT_PB_3 25 421 691/23 197 293
Antibodies	polyclonal rabbit anti-ParB antibody derived from serum, affinity purified using recombinant S. venezuelae ParB
Peak calling parameters	bowtie2 end-to-end<br>MACS2 --nomodel -g 8.2e+6 --keep-dup all<br>normr - reads were calculated using 100 bp window
Data quality	Read files were checked with fastqc. Adapters were trimmed from reads by Fasteris.<br>MACS2: only peaks with fold > 2 and FDR < 0.05 were analyzed<br>normr: diffR function was used to normalize ChIP-seq reads, only regions with FDR < 0.001 were analyzed
Software	data collection (provided by Fasteris)<br>NextSeq Control Software 4.2.0 112<br>RTA 2.11.3<br>bcl2fasta2. 17 v2 17.1.14<br><br>data analysis: bowtie2 2.5.2,<br>samtools 1.19.2,<br>MACS2 2.2.9.1,<br>R package normr 1.30.0
